# Challenges to code status discussions for pediatric patients

**DOI:** 10.1371/journal.pone.0187375

**Published:** 2017-11-02

**Authors:** Katherine E. Kruse, Jason Batten, Melissa L. Constantine, Saraswati Kache, David Magnus

**Affiliations:** 1 Stanford Center for Biomedical Ethics, Stanford University School of Medicine, Stanford, California, United States of America; 2 Division of Pediatric Critical Care Medicine, Department of Pediatrics, Stanford University School of Medicine, Stanford, California, United States of America; 3 Division of Health Policy and Management, University of Minnesota School of Public Health, Minneapolis, Minnesota, United States of America; University of Miami School of Medicine, UNITED STATES

## Abstract

**Objectives:**

In the context of serious or life-limiting illness, pediatric patients and their families are faced with difficult decisions surrounding appropriate resuscitation efforts in the event of a cardiopulmonary arrest. Code status orders are one way to inform end-of-life medical decision making. The objectives of this study are to evaluate the extent to which pediatric providers have knowledge of code status options and explore the association of provider role with (1) knowledge of code status options, (2) perception of timing of code status discussions, (3) perception of family receptivity to code status discussions, and (4) comfort carrying out code status discussions.

**Design:**

Nurses, trainees (residents and fellows), and attending physicians from pediatric units where code status discussions typically occur completed a short survey questionnaire regarding their knowledge of code status options and perceptions surrounding code status discussions.

**Setting:**

Single center, quaternary care children’s hospital.

**Measurements and main results:**

203 nurses, 31 trainees, and 29 attending physicians in 4 high-acuity pediatric units responded to the survey (N = 263, 90% response rate). Based on an objective knowledge measure, providers demonstrate poor understanding of available code status options, with only 22% of providers able to enumerate more than two of four available code status options. In contrast, provider groups self-report high levels of familiarity with available code status options, with attending physicians reporting significantly higher levels than nurses and trainees (p = 0.0125). Nurses and attending physicians show significantly different perception of code status discussion timing, with majority of nurses (63.4%) perceiving discussions as occurring “too late” or “much too late” and majority of attending physicians (55.6%) perceiving the timing as “about right” (p<0.0001). Attending physicians report significantly higher comfort having code status discussions with families than do nurses or trainees (p≤0.0001). Attending physicians and trainees perceive families as more receptive to code status discussions than nurses (p<0.0001 and p = 0.0018, respectively).

**Conclusions:**

Providers have poor understanding of code status options and differ significantly in their comfort having code status discussions and their perceptions of these discussions. These findings may reflect inherent differences among providers, but may also reflect discordant visions of appropriate care and function as a potential source of moral distress. Lack of knowledge of code status options and differences in provider perceptions are likely barriers to quality communication surrounding end-of-life options.

## Introduction

In the context of serious or life-limiting illness, pediatric patients and their families are faced with difficult decisions about withholding therapies, such as cardiopulmonary resuscitation (CPR). After discussion with the family, a physician may change a patient’s code status from full code to a resuscitation-limiting code to avoid inappropriate measures in the event of clinical deterioration. Limited published research finds providers are often reluctant to have discussions with families about code status options [1]. Additionally, when these discussions occur, they may occur too late in the course of disease to optimize end-of-life care.

Failure to have these discussions, or to have them early enough in the course of disease may prevent the patient and family from meaningfully directing their end-of-life care in a way that is consistent with their desires. It is traditionally the responsibility of the physician to initiate these discussions with patients and families. Pediatric studies have demonstrated that level of training correlates with provider discomfort in having challenging conversations with families [2–6]. It is expected that increased medical experience and training results in greater competence and level of comfort in discussing end-of-life issues, such as code status options, with several studies suggesting pediatric trainees have deficits in this area [2, 5–9].

Code status orders are one way to inform end-of-life medical decision making. Institutions have different code status options built into the electronic health record (EHR), with some having a small number of options, while other have adopted more nuanced options. This study was motivated by a recent expansion of available code status options at an academic pediatric institution. The new policy maintained the preexisting option of Partial Code, but expanded the Do-Not-Resuscitate (DNR) to Do-Not-Resuscitate/Do-Not-Intubate (DNR/DNI), Do-Not-Resuscitate/Do-Not Escalate (DNR/DNE), and Do-Not-Resuscitate/Comfort Care only (DNR/C) for a total of 4 code status options (in addition to Full Code). The intent of this study is to evaluate the extent to which providers have knowledge of available code status options and to explore the association of provider role with (1) knowledge of code status options, (2) perception of timing of code status discussions, (3) perception of family receptivity to code status discussions, and (4) comfort carrying out code status discussions.

## Materials and methods

### Overview

This is a cross-sectional survey study conducted in a large, academic, quaternary care children’s hospital. Survey data were collected from nurses, trainees (residents and fellows), and attending physicians in pediatric units where code status discussions typically occur: intensive care unit (ICU), cardiovascular ICU, neonatal ICU, and oncology. Content, format, and question structure are described below. Surveys did not contain any personally identifiable information or linkage to personally identifiable information. This study was reviewed and deemed exempt by the Stanford Institutional Review Board.

### Sampling and recruitment

The study sample is nurses, trainees, and attending physicians who were actively providing care in the pediatric ICU, cardiac ICU, neonatal ICU, and oncology units of the study institution. Eligible providers were recruited to complete surveys during formal (monthly divisional faculty/nurse meetings and trainee educational series) and informal (team huddles and approaching eligible providers throughout units) settings using a drop-off method. A total sample size of 157 is adequate to detect a difference of 0.10 in 2-way comparisons of provider role mean knowledge score with alpha = 0.05 and power = 0.80 [10]. Of eligible providers (approximately n = 530), sequential enrollment was used to enroll study participants. Recruitment and data collection occurred in October 2015.

### Survey design

Question and survey design adhered to the tailored design method [11] to provide design clarity to minimize respondent burden and improve survey and item non-response. Questions were developed to assess provider familiarity with the institutional code status options, as defined by both policy and EHR system, which had adopted more nuanced options the year prior. Additionally, provider perceptions of timing and family receptivity to code status discussions and comfort carrying out these discussions with families were evaluated.

Knowledge of code status options was assessed using an open-ended question with the example of “Full Code,” with respondents asked to list all available code status options. All other questions were close-ended with response options provided, including Likert-type response and semantic differential rating scales. The final survey instrument contained 10 items and time to complete was approximately 1 minute.

### Data analysis

The objective knowledge variable, indicating number of code status options correctly identified, is coded as a count variable, with possible values of 0 (no correct identification of any code status options) to 4 (correct identification of all 4 code status options). Bivariate associations between provider role groups and variables expected to differ across group are evaluated using ANOVA or chi-square (or Fishers exact where cell sizes < 5), with paired t-tests for difference of means. All statistical analyses were performed using SAS 9.4.

## Results

Of the roughly 530 eligible providers (RN = 420, trainee = 65 and attending physician = 45), 292 were approached for participation during the study period. Of these 292 providers, 263 completed surveys, including 203 nurses, 31 trainees, and 29 attending physicians, with an overall response rate of 90%. The majority of respondents are nurses, with fairly equivalent proportion of trainee and attending physician respondents. The sample provider ratio is comparable to the hospital provider ratio.

### Knowledge of code status options

There was no significant difference between providers in actual knowledge of code status options, which was consistently low (Table 1, p = 0.6041). Only 22% of providers were able to enumerate more than two of four available code status options. There was no identifiable pattern in the range of errors, some recalling one or both previous policy options (“DNR” or “Partial”), others listing one or more of the current policy options (“DNR/DNI,” “DNR/DNE,” and “DNR/C”), while others entering specific interventions (“No cardiac medications, “No ECMO,” etc).

**Table 1:** Differences in self-reported familiarity and objective knowledge of code status options across providers.

	Nurse N = 203 (77.2%)	Trainee N = 31 (11.8%)	Attending Physician N = 29 (11.0%)	Total
Knowledge of code status options: number of correct code status options indicated (p = 0.6041)				
0	51 (25.1)	5 (16.1)	6 (20.7)	62 (23.6)
1	73 (36.0)	8 (25.8)	8 (27.6)	89 (33.8)
2	39 (19.2)	10 (32.3)	6 (20.7)	55 (20.9)
3	28 (13.8)	6 (19.4)	6 (20.7)	40 (15.2)
4	12 (5.9)	2 (6.5)	3(10.3)	17 (6.5)
Self-reported familiarity with code status options (p = 0.0125)				
Not Familiar at All	8 (4%)	2 (6.5%)	0	10 (3.9)
Not Very Familiar	36 (18.0%)	6 (19.4%)	5 (17.2%)	47 (18.1)
Somewhat Familiar	121 (60.5%)	22 (71.0%)	12 (41.4%)	155 (59.6)
Very Familiar	35 (17.5%)	1 (3.2%)	12 (41.4%)	48 (18.5)

In contrast to the objective knowledge measure, over 78% of all providers self-report being somewhat or very familiar with institutional code status options, with attending physicians perceiving themselves to have greater familiarity than nurses or trainees (Table 1, p = 0.0125).

### Comfort with code status discussions

**Fig 1:**
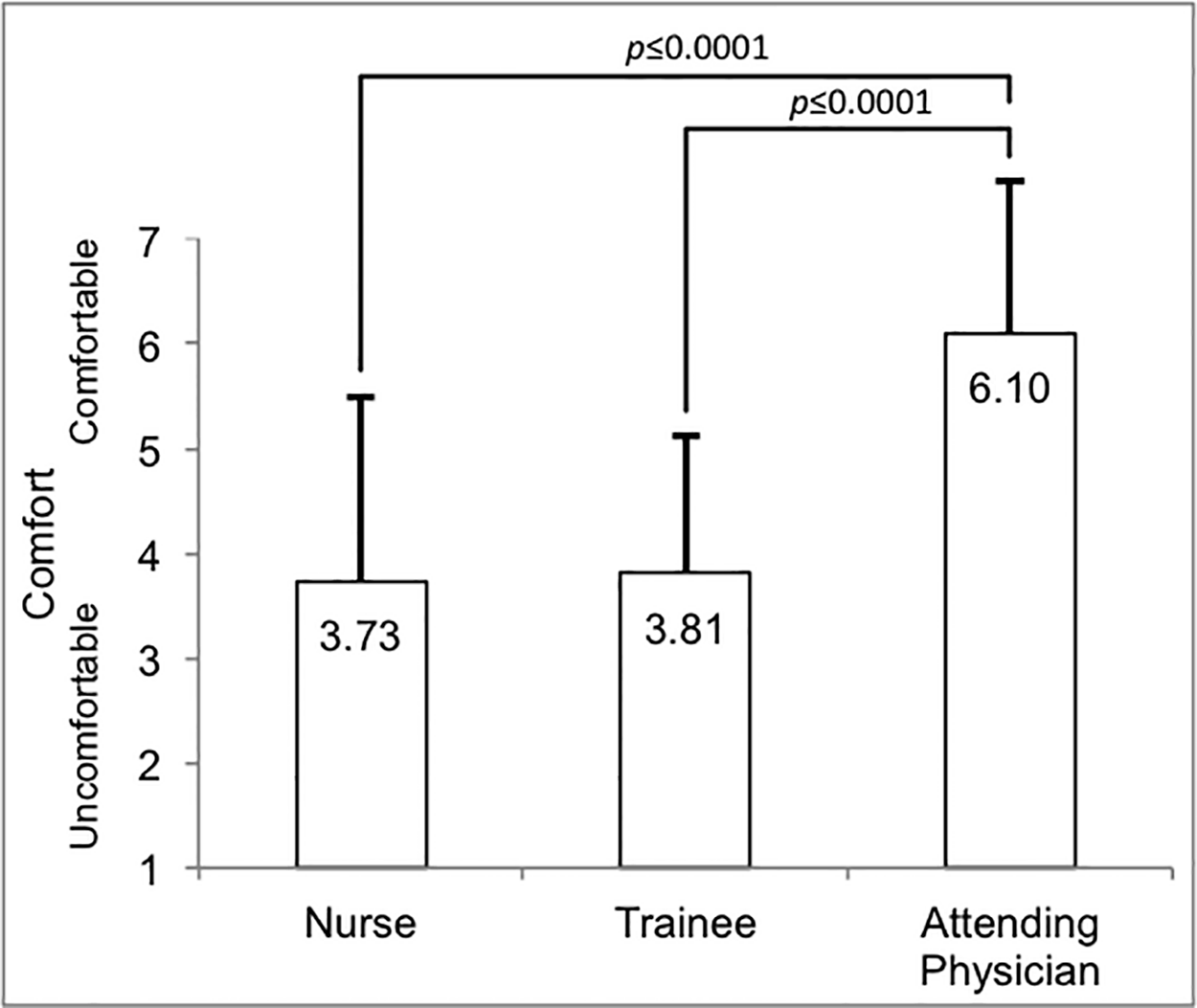
Provider comfort with code status discussions.

Attending physicians report higher comfort with having code status discussions with patients and families than do nurses or trainees (Fig 1, F = <0.0001; p≤0.0001), with 59.2% of attending physicians selecting the highest value for comfort. The difference between nurses and trainees is not statistically significant (p = 0.8085).

### Perception of family receptivity to code status discussions

**Fig 2:**
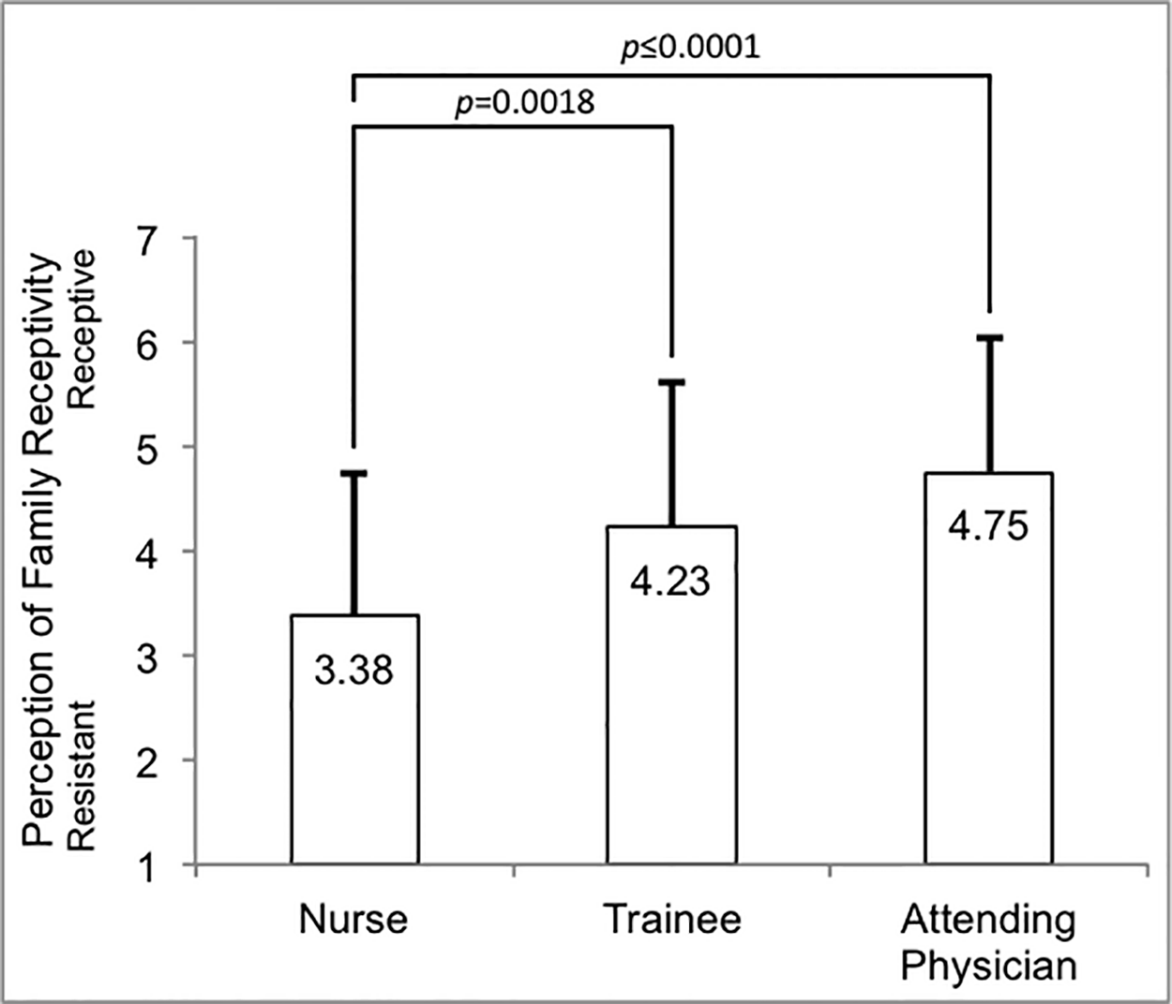
Provider perception of family receptivity to code status discussions.

Trainees perceive families to be more receptive to code status discussions than do nurses (Fig 2, p = 0.0018), and attending physicians also perceive families to be more receptive to code option discussions than do nurses (p<0.0001). The difference between attending physicians and trainees is not statistically significant (p = 0.1396).

### Perception of code status discussion timing

No providers believe discussions occur “much too soon”, and only a very small percentage of trainees (3%) believe discussions occur “too soon.” Nurses and attending physicians show significantly different perceptions of code status discussion timing (Fisher’s exact p<0.0001)), with the majority of nurses (63.4%) perceiving discussions as happening “too late” or “much too late,” and the majority of attending physicians (55.6%) perceiving them as “about right.” No attending physicians believe that code status discussions occur “much too late.” Trainees fall between nurses and attending physicians and do not differ significantly from either group.

## Discussion

Discussing resuscitation preferences in the setting of life-limiting illness helps providers make informed decisions that best support patients and families. Institutions commit resources to develop and vet code status policies and then implement them in their EHR to create a framework that supports decision-making for patients with life-limiting illness.

Despite hospital-wide initiatives and palliative care services in many adult institutions, hospitalized patients at risk of cardiopulmonary arrest often have not had discussions about code status [12] or documentation of such discussions in their medical records [13–15]. The SUPPORT study reported that only half of patients who preferred not to be resuscitated had a DNR order in their medical record [16]. To our knowledge, there are no pediatric studies looking at the occurrence of code status discussions or documentation in the EHR record, or instances where the EHR code status order conflicts with patient and/or parent wishes. The current study uses surveys of provider knowledge and perception to explore potential challenges associated with code status discussions that may underlie these failures.

Hospital code status policies are intended to improve communication and end-of-life treatment decisions [17]. Though all provider groups exhibit high levels of confidence in their familiarity with available code status options (particularly attending physicians), they actually have poor understanding of their options, despite educational interventions and existence of these options in the EHR system. Lack of knowledge of code status options may be a barrier to quality communication surrounding end-of-life options.

In our study, very few providers believe code status discussions occur too early, and a large percentage of providers believe they occur too late. This is consistent with previous pediatric studies showing that most clinicians believe code status discussions take place later than they should [1, 18]. Often these discussions occur merely hours before a child’s death [19]. One adult study of oncologists revealed discussions occurred only if prompted by the patient’s family [20]. The findings in this and other studies may reflect inherent differences among providers, such as variations in level of autonomy, but may also reflect discordant visions of appropriate care. In any case, these differences suggest a need for improved communication among various types of providers.

Lapses in communication and differences in understanding of when aggressive resuscitation measures are appropriate is likely to contribute to moral distress, which ultimately has a negative effect on patient care. Solomon et al (2005) showed that more than two-thirds of pediatric physicians and nurses agree that they “are saving children who should not be saved” [21]. However, while their study found that physicians were more likely to agree with this statement than nurses, our study found that nurses were more likely than attending physicians to report that discussions were happening “too late.” We speculate this may reflect a greater degree of moral distress among nurses in our population. Additionally, these differences in perception suggest that one challenge to improving quality of communication regarding code status is the divergence in perception about the appropriateness of aggressive treatment measures amongst various team members.

In critical illness, families are often forced to process information given in brief encounters laden with medical jargon. Providers are likewise challenged to balance conflicting obligations to disclose poor prognosis and continue to provide hope [22]. Adding to these challenges, different providers play different roles in communication with families. For example, families may confide their desires and fears to bedside nurses more often than they do to physicians [23]. Such differences in role may explain our finding that nurses perceive families as less receptive to code status discussions as compared to physicians. These differences may not be universal, as nurses in another study were least likely to believe that code status discussions were difficult and rather found them gratifying, unlike physicians [24]. Nevertheless, the feeling that families are not receptive to discussions about limiting inappropriately aggressive measures may be a source of moral distress.

We also found that nurses and trainees are less comfortable than attending physicians in having code status discussions. Nurses and physicians receive little to no structured or bedside training in code status discussions, leaving them underprepared [1]. Even with increased attention on communication surrounding end-of-life, pediatric trainees may still feel underprepared and lack the confidence necessary to engage in these discussions [2–9].

In summary, we observe dramatic differences in perspective by professional role, most notably between attending physicians and nurses. Attending physicians were more likely than nurses to report familiarity with institutional policy, perceive that discussions are timely, express comfort having discussions, and perceive that families are receptive to discussions. Trainees were consistently intermediate between nurses and attending physicians, agreeing more with nurses on some topics and more with attending physicians on others. These findings may reflect inherent differences among providers by role, including differences in power and autonomy, time spent at bedside, or professional education.

Nevertheless, our findings of substantial differences by provider role may also reflect discordant visions among the medical team of appropriate care and communication for seriously ill pediatric patients. This is unfortunate, since each member of the medical team is valuable and should contribute unique support to patients and families [25]. Furthermore, an interdisciplinary approach improves communication of sensitive information between providers and families [26– 27]. Our findings also suggest a potential source of moral distress among team members. Nurses, in particular, are more likely to perceive discussions as happening “too late” and see families as unreceptive to discussions and may therefore bear an undue share of moral distress. Moral distress has important negative effects on providers, their functioning as an interdisciplinary team, and patient safety [28–30].

### Limitations

Our study was conducted at a single quaternary children’s hospital, which may not reflect the culture and perspectives found in all pediatric institutions and limits the generalizability of these findings. Additionally, our data represents the provider’s perspective only; we did not assess patient and parental perspectives regarding code status discussions. Another limitation is that data is pooled from four pediatric units as there was insufficient power to analyze units individually. There may be differences among services that we were unable to capture. For example, when comparing intensivists with oncologists, the latter often have longer-term relationships with patients and families that cross over outpatient encounters. Additionally, there is heterogeneity amongst trainees; one would expect an intern with less experience and training to have a different perspective than a third-year critical care fellow. Lastly, the vast majority of our respondents were nurses, with relatively few trainees and attending physicians. However, this proportion is an accurate representation of the proportion of nurses to attending physicians and trainees at the hospital in which this study was conducted.

## Conclusions

Our findings reveal that providers have poor understanding of existing code status options, even though most believe themselves to be familiar with them. Fundamental lack of knowledge represents a barrier for carrying out informed code status discussions; educational interventions may be required to address this knowledge gap. In addition, there are significant differences by provider role in perceptions of code status discussion timing and family receptivity to these discussions, as well as differences in comfort with having them. Future research may build on these findings and overcome some of the above limitations by (a) quantitatively assessing knowledge of and perceptions surrounding code status options at other institutions and (b) qualitatively exploring provider and patient/family perspectives surrounding code status discussions, which would also serve to illuminate views about the optimal timing of these discussions.

## Supporting information

S1 File Survey. (PDF)

S2 File Code status data. (XLSX)
