# Supplementary material for: Challenges to code status discussions for pediatric patients
Source: PLoS One. 2017 Nov 2;12(11):e0187375. doi: 10.1371/journal.pone.0187375 (PMC5667871; doi:10.1371/journal.pone.0187375)
Supplement: S1 File — (PDF) [file pone.0187375.s001.pdf]

Date __/__/____

Q1. What is your primary Division at LPCH?

1☐ PICU
2☐ CV-ICU
3☐ NICU
4☐ Oncology
5☐ Hospitalist

Q2. Which of the following best describes your current professional role at LPCH?

1☐ Respiratory Therapist
2☐ Fellow
3☐ Resident
4☐ Nurse
5☐ Social Worker
6☐ Attending
7☐ Advanced Nurse Practitioner
8☐ Physician Assistant

Q3. How long have you been in your current position at LPCH? ___________

Q4. How familiar are you with code status options that are available, other than Full Code, at LPCH?

1☐ Very Familiar
2☐ Somewhat Familiar
3☐ Not Very Familiar
4☐ Not Familiar at All

Q5. As far as you know, what are the code options that are available at LPCH?

Code	Full	Code		Code	
Code		Code		Code	
Code		Code		Code	

Q6. Please circle the number that best corresponds to your level of comfort having discussions with your patient's families about changing code status.

1	2	3	4	5	6	7
COMFORTABLE						UNCOMFORTABLE

Q7. What is your opinion about the timing of discussions about changing code status that have taken place in your Division with families over the last 6 months? Have the discussions with families taken place sooner than they should have, at about the right time, or later than they should have?

1☐ Much too soon
2☐ Too soon
3☐ About the right time
4☐ Too late
5☐ Much too late

Q8. In your experience over the last 6 months, how receptive or resistant have families been to discussing a change in code status?

1	2	3	4	5	6	7
RECEPTIVE						RESISTANT

Q9. In the last 6 months, how often would you say you have had conversations with other staff in your Division about a potential change in code status for a patient?

1☐ Never
2☐ Rarely
3☐ Sometimes
4☐ Often

Q10. Within the last 6 months, have you experienced any of the following type meetings, educational interventions, or specific inclusion of **expanded code status options** at LPCH? Please check all that apply.

1☐ Division lecture, seminar or presentation
2☐ Rounding checklist that includes discussion of code status
3☐ Safety rounds or huddles that include discussion of code status
4☐ EMR prompt specific to code status
5☐ Hospital-wide lecture, seminar or presentation e.g., Grand Rounds
6☐ Other. Please describe. ________________________________
